# Effects of Emissions from Heated Tobacco Products and Reference Cigarettes on Gene Expression and Mitochondrial Function in Human Lung Epithelial BEAS-2B Cells †

**DOI:** 10.3390/antiox14121404

**Published:** 2025-11-25

**Authors:** Suin Park, Miil Kim, Wei Jin, Ji Yun Yeo, Jae-Hyeong Kim, Yoon-Seok Seo, Jung-Min Park, Jinhee Kim, Min-Seok Kim, Donghyun Kim, Ok-Nam Bae, Choongho Lee, Moo-Yeol Lee

**Affiliations:** 1 BK21 FOUR Team and Integrated Research Institute for Drug Development, College of Pharmacy, Dongguk University, Goyang 10326, Gyeonggi-do, Republic of Korea; 2 Center for Respiratory Safety Research, Korea Institute of Toxicology, Jeongeup 56212, Jeonbuk-do, Republic of Korea; 3 College of Pharmacy, Keimyung University, Daegu 42601, Republic of Korea; 4 College of Pharmacy, Institute of Pharmaceutical Science and Technology, Hanyang University, Ansan 15588, Gyeonggi-do, Republic of Korea

**Keywords:** heated tobacco products, cigarettes, cytotoxicity, mitochondria, whole cigarette smoke condensates

## Abstract

Heated tobacco products (HTPs) are marketed as lower-risk alternatives to conventional cigarettes; however, their toxicological impacts remain insufficiently characterized. This study evaluated the effects of HTP emissions on gene expression and mitochondrial function in comparison with conventional cigarettes. Whole cigarette smoke condensates (WCSCs), comprising both gas and particulate phases, were prepared from three commercially available HTPs and from 3R4F reference cigarettes. Human lung epithelial BEAS-2B cells were exposed to WCSCs at 3 μg nicotine/mL for 24 h, followed by transcriptome profiling using RNA sequencing. Principal component analysis demonstrated that HTP-WCSCs induced weaker gene expression changes than 3R4F-WCSC, with only modest variation among HTPs. Gene set enrichment analysis revealed that both HTP- and 3R4F-WCSCs significantly downregulated oxidative phosphorylation (OXPHOS)–related pathways, indicating potential mitochondrial impairment. Functional assays confirmed that both exposures elevated mitochondrial reactive oxygen species (ROS), while mitochondrial morphology, ATP production, membrane potential, and cytosolic ROS were largely unaffected. Collectively, these results show that although HTP emissions elicit weaker transcriptomic perturbations than conventional cigarette emissions, both converge on mitochondrial targets by suppressing OXPHOS gene expression and increasing mitochondrial ROS. Mitochondrial dysfunction may therefore represent a common mechanism underlying tobacco product toxicity.

## 1. Introduction

Heated tobacco products (HTPs), also referred to as heat-not-burn cigarettes, are devices that heat tobacco at lower temperatures than conventional cigarettes [1]. Typically, HTPs heat tobacco to below 350 °C to generate an aerosol rather than smoke, whereas conventional cigarettes combust tobacco at temperatures approaching 900 °C [2]. This technology is intended to reduce the harmful effects of cigarette smoking, primarily through reduced tar yields. Since their commercial introduction in the 2010s, HTPs have rapidly gained popularity and market share, often promoted as potentially less harmful alternatives to conventional cigarettes [3,4]. However, their widespread adoption has also raised public health and regulatory concerns regarding their true health impact [5].

Several studies suggest that HTPs emit lower levels of toxicants than conventional cigarettes [6,7,8], but the evidence remains incomplete and sometimes contradictory. Lower heating temperatures can decrease toxicant formation [9], and in 2020, the U.S. Food and Drug Administration authorized certain HTPs as modified-risk tobacco products based on reduced exposure to selected harmful chemicals [10]. Despite these advantages, HTPs still release a range of potentially harmful constituents, including nicotine and other toxicants, some of which are absent from conventional cigarette smoke [2]. Their toxicities and underlying mechanisms remain insufficiently defined, and a comprehensive toxicological profile is required to accurately assess their potential health risks.

Transcriptomic profiling provides a powerful approach to investigate the toxicological characteristics of HTPs. Alterations in gene expression can reveal cellular and molecular responses to toxicant exposure and identify disrupted biological pathways [11,12,13,14]. Advances in next-generation sequencing technologies now permit comprehensive transcriptomic analyses, also enabling comparisons of gene expression changes induced by HTPs versus conventional cigarettes. Previous RNA sequencing studies have shown that HTPs generally cause fewer transcriptomic alterations than conventional cigarettes [15,16]. Nevertheless, important limitations persist. For example, exposure levels have not always been clearly defined, and functional validation of transcriptomic changes has often been insufficient [17,18,19]. Moreover, comparative studies using conventional cigarettes as controls are essential to strengthen the toxicological interpretation of transcriptomic findings.

In this study, we investigated the effects of HTP emissions on gene expression in a cultured cell system. Whole cigarette smoke condensates (WCSCs) were prepared from three commercially available HTPs as well as the 3R4F reference cigarette, and exposures were carried out in human bronchial epithelial BEAS-2B cells, a relevant model since the respiratory epithelium is a primary site of inhalation exposure [20]. Transcriptomic alterations were assessed by means of RNA sequencing and bioinformatic analysis. Our goal was to characterize transcriptomic responses to HTP exposure at lower, clinically relevant levels compared with those used in earlier studies, thereby more closely reflecting realistic smoker exposure [18,21,22]. In addition, based on the transcriptomic findings, we evaluated mitochondrial functions, focusing on oxidative phosphorylation (OXPHOS) and reactive oxygen species (ROS) formation, to determine whether transcriptomic alterations were accompanied by functional changes in mitochondria. This combined approach provides novel insights into mitochondrial involvement in HTP-induced toxicity.

## 2. Materials and Methods

### 2.1. Reagents

Lil [Korea Tomorrow & Global (KT&G), Daejeon, Republic of Korea], IQOS (Philip Morris International, Neuchâtel, Switzerland), and Glo (British American Tobacco, London, UK) were purchased from a local cigarette retailer. 3R4F reference cigarettes were obtained from the Center for Tobacco Reference Products, University of Kentucky (Lexington, KY, USA). MitoTracker Red CMXRos, MitoSOX Red, dihydrorhodamine 123 (DHR123), tetramethylrhodamine methyl ester (TMRM), 10-acetyl-3,7-dihydroxyphenoxazine (Amplex Red), 1,2-bis(2-aminophenoxy)ethane-*N*,*N*,*N*’,*N*’-tetraacetic acid acetoxymethyl ester (BAPTA-AM), bicinchoninic acid (BCA) protein assay kit, and cell culture reagents, including Roswell Park Memorial Institute (RPMI) 1640 medium, fetal bovine serum (FBS), penicillin–streptomycin, and Hank’s balanced salt solution (HBSS), were obtained from Thermo Fisher Scientific (Waltham, MA, USA). Primary antibodies against dynamin-related protein 1 (Drp1), phospho-Drp1 (Ser616), mitofusin-1 (Mfn1), and mitofusin-2 (Mfn2), as well as horseradish peroxidase (HRP)-conjugated anti-rabbit and anti-mouse IgG secondary antibodies, were acquired from Cell Signaling Technology (Danvers, MA, USA). Carbonyl cyanide m-chlorophenyl hydrazone (CCCP), HRP, and crystal violet were obtained from Sigma-Aldrich (St. Louis, MO, USA). Additional reagents were as follows: Complete protease inhibitor cocktail, PhosSTOP phosphatase inhibitor cocktail, and X-tremeGENE HP DNA transfection reagent (Roche Diagnostics, Mannheim, Germany); Seahorse Mitostress test kit and XF RPMI medium (Agilent, Santa Clara, CA, USA); pCS2+MLS-HyPer7 plasmid and pLJM1 lentiviral vector (Addgene, Cambridge, MA, USA); Premix WST-1 (Takara Bio, Kusatsu, Japan); RNeasy kit (Qiagen, Hilden, Germany); NEBNext Ultra II Directional RNA Library Prep kit (New England Biolabs, Ipswich, MA, USA); Poly(A) RNA selection kit (Lexogen, Vienna, Austria); Immobilon Western chemiluminescent HRP substrate (Donginbiotech, Seoul, Republic of Korea); CellTiter-Glo luminescent cell viability assay kit (Promega, Madison, WI, USA); and radioimmunoprecipitation assay (RIPA) buffer (Biosolution, Suwon, Republic of Korea). All other chemicals were of the highest available purity and obtained from standard commercial suppliers.

### 2.2. Preparation of WCSCs

WCSCs were prepared as previously described [23], with minor modifications. Cigarettes were conditioned at 22 ± 1 °C and 60 ± 2% relative humidity for 48 h in accordance with ISO 3402 standards [24]. Emissions were generated from 80 sticks of HTPs (Lil, IQOS, and Glo) using their respective heating devices or from 15 sticks of 3R4F reference cigarettes using an SG-300 cigarette smoke generator (Sibata, Saitama, Japan), following the Health Canada Intense smoking protocol: 55 mL puff volume, 2-s puff duration, 30-s puff interval, and 100% filter vent blocking [25].

Particulate matter was trapped on a 44 mm Cambridge filter pad (GE Healthcare, Little Chalfont, UK), while the remaining gas phase was absorbed into 50 mL of Dulbecco’s phosphate-buffered saline at a flow rate of 1.65 L/min for 5 min using an impinger and sampling pump, yielding the cigarette smoke extract (CSE). Particulate matter retained on the filter pad was extracted with 0.5 mL methanol, evaporated under nitrogen, and resuspended in dimethyl sulfoxide to generate the total particulate matter (TPM). WCSCs were prepared by combining TPM and CSE and stored at −80 °C until use.

The measured TPM concentrations were 24.4, 27.5, 29.0, and 20.6 mg/mL for Lil-, IQOS-, Glo-, and 3R4F-WCSCs, respectively, with corresponding nicotine concentrations of 1.81, 2.45, 2.26, and 1.10 mg/mL (Table 1). Nicotine concentrations were determined using a GC-2010 gas chromatography system (Shimadzu, Kyoto, Japan) according to established protocols [23].

### 2.3. Cell Culture

The human bronchial epithelial cell line BEAS-2B was obtained from the American Type Culture Collection (Manassas, VA, USA) and cultured in RPMI 1640 medium supplemented with 10% FBS, 100 U/mL penicillin, and 100 µg/mL streptomycin. Cells were maintained at 37 °C in a humidified incubator with 5% CO_2_ and subcultured at 80–90% confluence.

### 2.4. Assessment of Cell Viability and Growth

Cell viability and growth were assessed using the WST-1 and crystal violet staining assays [26,27]. For the WST-1 assay, cells were treated with WCSCs for 24 or 48 h, followed by incubation in 200 µL phenol red-free medium containing 20 µL Premix WST-1 for 1 h at 37 °C. For the crystal violet staining assay, WCSC-treated cells were fixed with 4% paraformaldehyde, stained with 0.1% crystal violet, washed, and dissolved in 1% sodium dodecyl sulfate (SDS). Absorbance was measured at 450 nm for the WST-1 assay and at 590 nm for the crystal violet assay using a SpectraMax M3 microplate reader (Molecular Devices, San Jose, CA, USA).

### 2.5. RNA Isolation and Library Preparation

Total RNA was extracted from WCSC-treated cells using the RNeasy kit. RNA integrity was evaluated using a 2100 Bioanalyzer (Agilent Technologies, Amstelveen, The Netherlands), and RNA concentration was determined with an ND-2000 spectrophotometer (Thermo Fisher Scientific).

Poly(A)+ RNA was enriched using the Poly(A) RNA Selection kit, and libraries were prepared using the NEBNext Ultra II Directional RNA Library Prep kit following the manufacturer’s instructions. RNA was fragmented, reverse-transcribed into cDNA, indexed with Illumina indexes 1–12, and amplified by polymerase chain reaction (PCR). Library quality was verified with TapeStation HS D1000 ScreenTape (Agilent), and quantification was performed using a library quantification kit and the StepOne real-time PCR system (Life Technologies, Carlsbad, CA, USA). Sequencing was carried out on a NovaSeq 6000 platform (Illumina, San Diego, CA, USA) with paired-end 100 bp reads.

### 2.6. RNA Sequencing Analysis

#### 2.6.1. Preprocessing

Raw read count matrices were imported into R (version 4.2.3; R Foundation, Vienna, Austria). Genes with low expression were filtered using the filter_genes function of the RNAseqQC package (version 0.2.1), retaining only those with a minimum of five counts in at least four replicates.

#### 2.6.2. Differential Expression Analysis

Differential expression analysis was performed using DESeq2 (version 1.38.3), with the control group as the reference. Count normalization was conducted using the median-of-ratios method, and log2 fold changes were stabilized with the apeglm shrinkage estimator. Genes were considered differentially expressed if they met the criteria of adjusted *p*-value < 0.05 and absolute log2 fold change ≥ 0.58 (~1.5-fold). Volcano plots were generated using ggplot2 (version 3.5.2) to visualize significantly up- and downregulated genes, with the most prominent candidates annotated.

#### 2.6.3. Principal Component Analysis (PCA) and Sample Correlation

Variance-stabilizing transformation (VST) and regularized log (rlog)-transformation of normalized counts were performed using DESeq2 (version 1.38.3) to reduce heteroscedasticity across samples. PCA was conducted on the VST-transformed expression matrix to visualize global transcriptomic variation among groups. Sample-to-sample correlations were computed from rlog-transformed counts using the Pearson correlation coefficient, and the resulting correlation matrix was visualized as a clustered heatmap generated with the ComplexHeatmap package (version 2.24.1).

#### 2.6.4. Gene Set Enrichment Analysis (GSEA)

Hallmark gene sets were retrieved from the Molecular Signatures Database (MSigDB) using the msigdbr package. Gene-level statistics were derived from DESeq2 results by ranking genes according to log2 fold change. For genes with duplicate symbols, the entry with the largest absolute log2 fold change was retained. GSEA was performed with the fgsea package (version 1.24.0), using 10,000 permutations and restricting gene set sizes to 10–500 members. Pathways with an adjusted *p*-value (false discovery rate, FDR) < 0.05 were considered significantly enriched. Normalized enrichment scores (NES) indicated the direction of regulation, with positive values representing upregulated pathways and negative values representing downregulated pathways. Enrichment results were visualized using ggplot2 by plotting all pathways ordered by |NES|, with significant pathways (FDR < 0.05) highlighted in blue.

### 2.7. Analysis of Mitochondrial Morphology

Cells were stained with 100 nM MitoTracker Red CMXRos for 30 min following 24 h of WCSC treatment. Images were acquired using an Eclipse Ti-U inverted microscope equipped with an S Fluor 100× oil-immersion objective lens (Nikon, Tokyo, Japan), a Lambda DG-4 filter changer with a xenon arc lamp (Sutter Instruments, Novato, CA, USA), and an Evolve 512 EMCCD camera (Photometrics, Tucson, AZ, USA). Fluorescence was detected at excitation and emission wavelengths of 475 and 560 nm, respectively. Images were analyzed using Meta Imaging System (version 7.7.6; Molecular Devices) and the Mitochondria Analyzer plugin in ImageJ (version 1.54f; National Institutes of Health, Bethesda, MD, USA) [28,29].

### 2.8. Immunoblot Analysis

Protein expression and phosphorylation of mitochondrial dynamics regulators were examined by Western blotting [30]. Cells were lysed in RIPA buffer supplemented with a Complete protease inhibitor cocktail and a PhosSTOP phosphatase inhibitor cocktail, and centrifuged at 16,200× *g* for 30 min. Supernatants were collected, and protein concentrations were determined using the BCA protein assay kit. Equal amounts of protein were separated by means of SDS–polyacrylamide gel electrophoresis, transferred to polyvinylidene difluoride membranes, probed with primary and HRP-conjugated secondary antibodies, and visualized using Immobilon Western chemiluminescent HRP substrate. Chemiluminescent signals were detected with the ChemiDoc XRS+ system and quantified using Image Laboratory software (version 3.0; Bio-Rad Laboratories, Hercules, CA, USA).

### 2.9. Measurement of Mitochondrial ROS (mtROS), Intracellular ROS, and Mitochondrial Membrane Potential

mtROS, intracellular ROS, and mitochondrial membrane potential were quantified using fluorescent probes coupled with flow cytometric analysis. Cells exposed to WCSCs for 24 h were stained with 5 μM MitoSOX Red for 20 min, 10 μM DHR123 for 30 min, or 100 nM TMRM for 30 min to detect mitochondrial ROS, intracellular ROS, and mitochondrial membrane potential, respectively. Stained cells were analyzed using a BD FACSAria III cell sorter (BD Biosciences, San Jose, CA, USA) and FlowJo software (version 7.6; BD Biosciences). Excitation/emission settings were 488/575 nm for MitoSOX and TMRM and 488/519 nm for DHR123.

mtROS was further assessed using the genetically encoded, mitochondria-targeted H_2_O_2_ sensor MLS-HyPer7. The construct was cloned into a pLJM1 vector and transfected into BEAS-2B cells using X-tremeGENE HP DNA transfection reagent. After 48 h, cells were seeded onto coverslips, treated with WCSCs for 24 h, and imaged with an Eclipse Ti-U inverted microscope (Nikon). Fluorescence images were acquired at excitation wavelengths of 405 and 480 nm with emission at 528 nm, and were analyzed using the Meta Imaging System (version 7.7.6; Molecular Devices).

Intracellular ROS production was further evaluated with the Amplex Red assay. Cells were incubated with 10 μM Amplex Red and 0.1 U/mL HRP in phenol red-free RPMI 1640 medium. Fluorescence was measured at excitation/emission wavelengths of 560/580 nm under kinetic mode for 60 min using a SpectraMax M3 microplate reader (Molecular Devices).

### 2.10. Measurement of ATP Levels

Cellular ATP levels were quantified using the CellTiter-Glo luminescent cell viability assay kit (Promega). Cells were exposed to WCSCs for 24 h, treated with CellTiter-Glo reagent, and stabilized for 10 min. Luminescence was measured using a Glomax Multi+ detection system (Promega).

### 2.11. Bioenergetic Profiling

Mitochondrial bioenergetic function was evaluated using the Seahorse XF HS Mini Analyzer with the Mitostress test kit. Cells were seeded into XFp miniplates (Agilent) at a density of 4.0 × 10^4^ cell/well, exposed to WCSCs for 24 h, and equilibrated in Seahorse XF RPMI medium. Oxygen consumption rate (OCR) and extracellular acidification rate were measured following sequential injection of electron transport chain modulators: oligomycin (1.0 μM), carbonyl cyanide-4-(trifluoro-methoxy) phenylhydrazone (FCCP, 1.0 μM), and rotenone/antimycin A (1.5 μM). Parameters, including ATP production-linked respiration, maximal respiration, proton leak, and non-mitochondrial respiration, were calculated. OCR values were normalized to protein content per well, as determined by the BCA protein assay kit.

### 2.12. Statistical Analysis

Data are expressed as mean ± standard error. Statistical analyses were performed using one-way analysis of variance followed by Dunnett’s post hoc test. All analyses were conducted with SigmaPlot (version 13; Systat Software, San Jose, CA, USA). A *p*-value < 0.05 was considered statistically significant.

## 3. Results

### 3.1. Overall Effects of HTP-WCSCs on Gene Expression in BEAS-2B Cells

**Figure 1:**
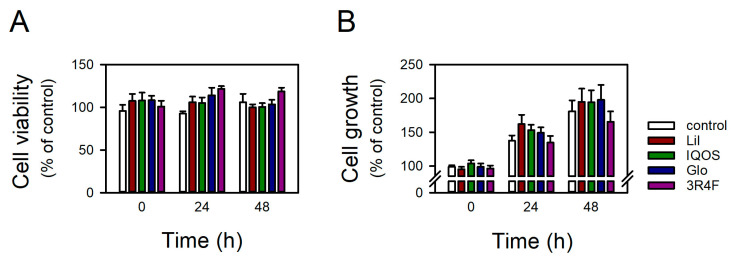
Effects of WCSCs on the viability and growth of BEAS-2B cells. (**A**) Cell viability and (**B**) growth were assessed using WST-1 and crystal violet assays. Cells were treated with 3 μg nicotine/mL WCSCs for up to 48 h. Values from the control group at 0 h were regarded as 100%. Data are presented as mean ± standard error (n = 6 for WST-1 assay; n = 5 for crystal violet assay).

To evaluate the impact of HTP aerosols on the respiratory epithelium, transcriptomic profiling was performed in BEAS-2B cells exposed to WCSCs. WCSCs were prepared from three commercially available HTPs (Glo, IQOS, and Lil) as well as from 3R4F reference cigarettes, and applied to cells at 3 μg nicotine/mL for 24 h. Transcriptome-wide analysis was conducted by RNA sequencing. This exposure concentration corresponds to the approximate nicotine level in a single puff of 3R4F cigarette smoke [31,32,33]. Cell viability and growth were unaffected for up to 48 h under these conditions (Figure 1).

**Figure 2:**
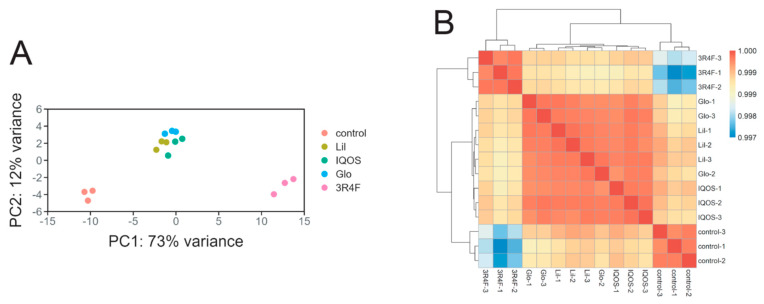
Transcriptomic profiles of cells exposed to HTP- and 3R4F-WCSCs. RNA sequencing was conducted on cells treated with WCSCs for 24 h. (**A**) PCA of normalized transcriptomes showing distinct clustering among experimental groups. (**B**) Heatmap of pairwise sample correlations based on variance-stabilized counts (n = 3 per group).

PCA revealed substantial changes in gene expression following WCSC exposure (Figure 2A). Alterations induced by HTP-WCSCs were less pronounced than those caused by 3R4F-WCSC, and transcriptomic profiles clustered more closely among HTP groups than between HTP- and 3R4F-exposed groups. Pairwise correlation analysis further supported this observation, demonstrating strong correlations within HTP-treated cells but weaker correlations between HTP- and 3R4F-exposed groups (Figure 2B).

**Figure 3:**
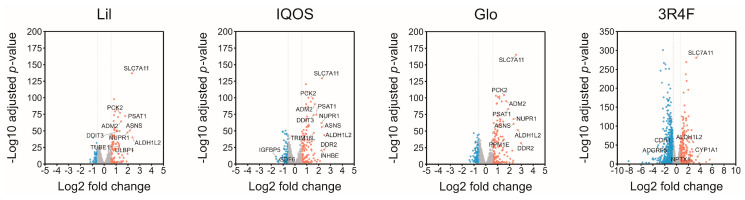
Differential gene expression induced by HTP- and 3R4F-WCSCs. Volcano plots depict DEGs in cells exposed to HTP- or 3R4F-WCSCs. The x-axis represents log2 fold change, and the y-axis denotes −log10 adjusted *p*-value. Genes meeting the criteria of log2 fold change ≥0.58 or ≤−0.58 and adjusted *p* < 0.05 are highlighted in red or blue, respectively. Genes that do not meet the fold-change cutoff (|log2 fold change| < 0.58) are displayed in gray, indicating non-significant expression changes. Please note that the y-axis scale of the 3R4F plot differs from those of the Lil, IQOS, and Glo plots.

Differentially expressed gene (DEG) analysis highlighted these differences in greater detail. Volcano plots revealed substantially fewer significantly altered genes in HTP-treated cells compared with 3R4F-treated cells (Figure 3). The numbers of altered genes at ≥1.5- and ≥2.0-fold changes were as follows: Lil, 127 and 24; IQOS, 171 and 33; and Glo, 225 and 40. In contrast, 3R4F-WCSC altered 1019 genes at ≥1.5-fold and 249 genes at ≥2.0-fold (Table 2). Overall, the greatest transcriptomic impact was observed with 3R4F-WCSC, followed by Glo, IQOS, and Lil, while differences among the HTP groups were relatively modest.

### 3.2. Enriched Biological Pathways Identified by GSEA

**Figure 4:**
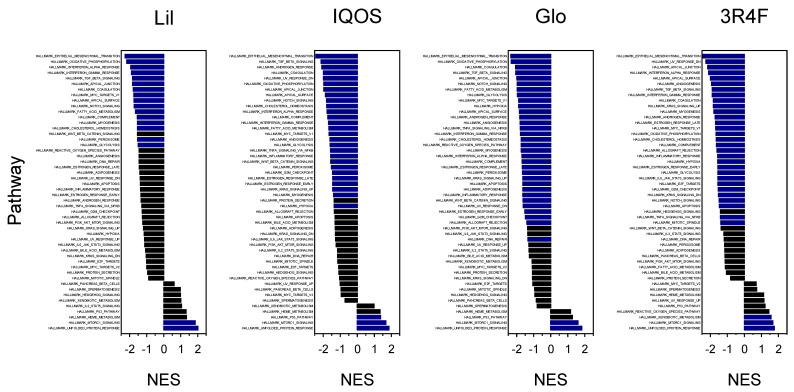
GSEA of Hallmark pathways affected by WCSCs. NES are shown for pathways in WCSC-exposed cells. Positive NES values indicate upregulation, whereas negative NES values represent downregulation. Pathways with adjusted *p* < 0.05 are highlighted in blue.

GSEA was performed using Hallmark gene sets from MsigDB to identify pathways perturbed by WCSC exposure (Figure 4). The top ten enriched pathways were ranked by NES for each exposure group. Across all conditions, epithelial–mesenchymal transition, TGF-β signaling, and apical junction pathways were consistently enriched. Notably, OXPHOS ranked among the top ten pathways in all HTP-treated groups but not in the 3R4F-WCSC group, suggesting a relatively greater emphasis on OXPHOS-related alterations in HTP exposures. In contrast, angiogenesis and KRAS signaling were more prominent in 3R4F-exposed cells, underscoring pathway-level distinctions between HTPs and conventional cigarettes (Table 3).

**Figure 5:**
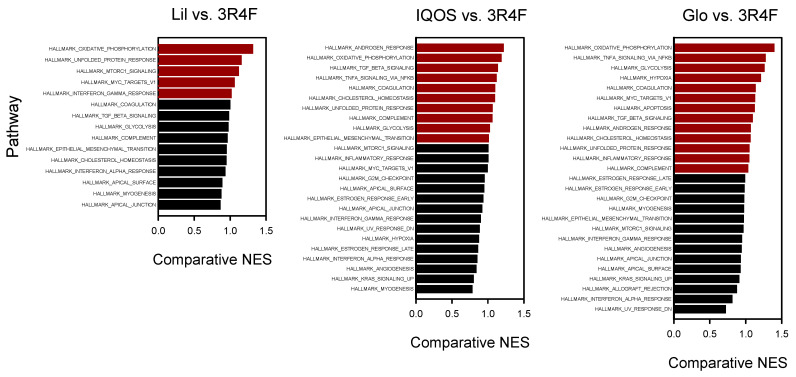
Comparative enrichment of Hallmark pathways by HTP- versus 3R4F-WCSCs. Comparative NES values illustrate relative pathway enrichment between HTP-WCSC- and 3R4F-WCSC-treated cells. Pathways more enriched in HTP-WCSC-exposed cells (comparative NES > 1) are shown in red.

Comparative GSEA between HTP- and 3R4F-treated cells revealed distinct patterns of pathway enrichment (Figure 5). Across all three HTPs, the OXPHOS pathway consistently exhibited the highest comparative NES, indicating a significantly stronger downregulation of mitochondrial respiratory gene sets relative to the 3R4F group. This trend was reproducible across HTPs, suggesting that suppression of mitochondrial oxidative metabolism represents a common transcriptomic feature specific to HTP exposure.

### 3.3. Changes in OXPHOS Pathway-Related Gene Expression

**Figure 6:**
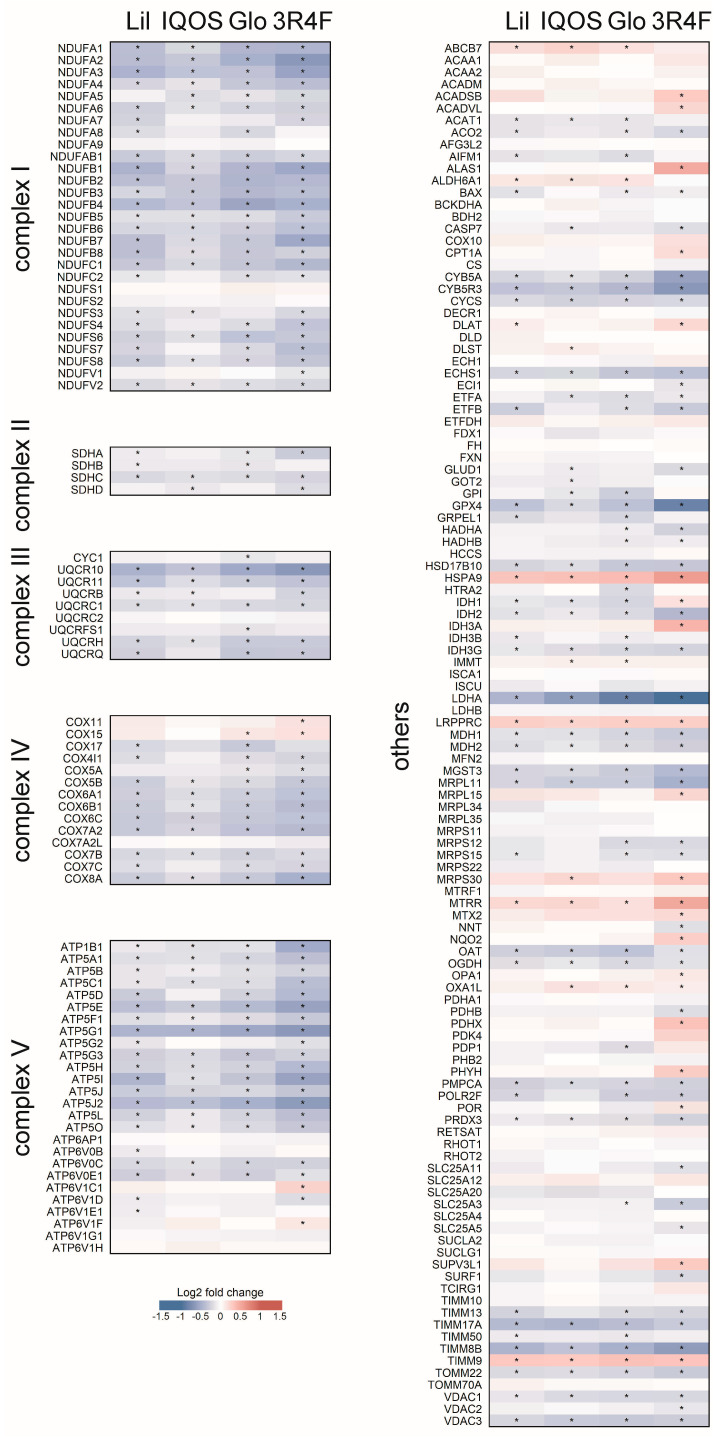
Downregulation of OXPHOS pathway genes by WCSCs. Heatmap of expression changes in genes associated with OXPHOS, categorized by mitochondrial respiratory complexes I–V and “others”. The color scale represents log2 fold change values. * adjusted *p* < 0.05.

The OXPHOS gene set comprises 197 genes encoding subunits of the mitochondrial respiratory complexes. Expression changes were visualized in a heatmap organized by complex, with additional genes categorized as “others” (Figure 6). WCSC exposure broadly downregulated genes across multiple complexes, indicating widespread suppression of mitochondrial respiratory capacity. Most of these changes represented statistically significant downregulation, while only a few genes were significantly upregulated. These transcriptomic signatures prompted subsequent experiments to determine whether WCSCs also induced functional alterations in mitochondrial physiology.

### 3.4. Effect of WCSCs on Mitochondrial Morphology and Dynamics

**Figure 7:**
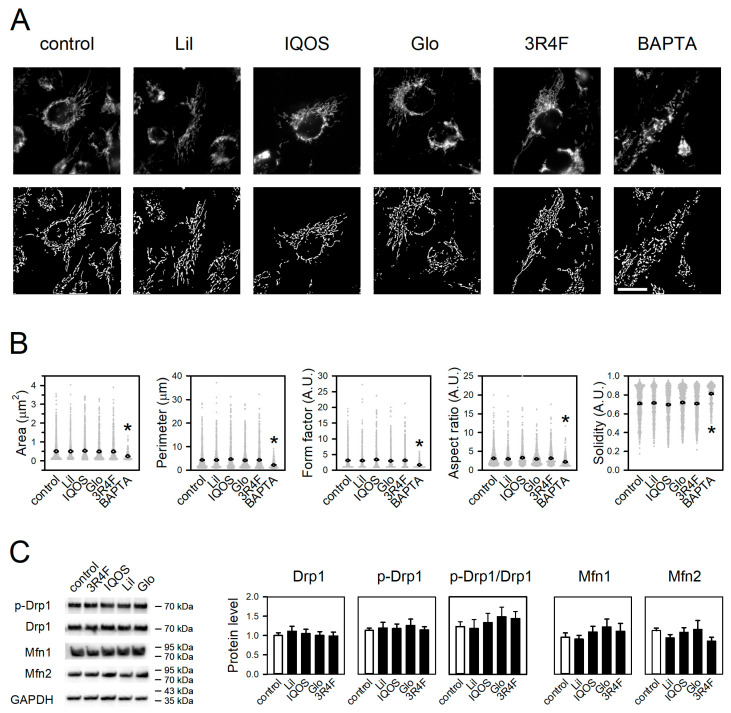
Effects of WCSCs on mitochondrial morphology and dynamics. Cells were treated with WCSCs for 24 h. (**A**) Representative images of mitochondria. Upper panels show raw images of mitochondria stained with MitoTracker Red CMXRos, and lower panels show processed images generated by ImageJ for quantitative morphology analysis. Scale bar = 5 μm. (**B**) Quantitative analysis of mitochondrial morphology across five parameters. (**C**) Effects of WCSCs on mitochondrial fission and fusion proteins. Phosphorylation of Drp1 at Ser616 and expression levels of Drp1, Mfn1, and Mfn2 were examined by immunoblotting. Representative blots and quantified band intensities normalized to untreated groups are shown. Values are mean ± standard error [n = 1028, 671, 739, 993, 740, and 489 for control and Lil-, IQOS-, Glo-, 3R4F-WCSC-, and BAPTA-AM-treated groups, respectively (**B**); n = 10, 8, 8, 8, and 9 for control and Lil-, IQOS-, Glo-, and 3R4F-WCSC-treated groups, respectively (**C**)]. * *p* < 0.05 vs. control.

The impact of WCSCs on mitochondrial morphology and dynamics was assessed in treated cells. Mitochondrial morphology was quantified by evaluating five parameters: area, perimeter, form factor, aspect ratio, and solidity [28,34]. Under control conditions, mitochondria typically exhibited an elongated morphology and formed interconnected networks throughout the cytoplasm (Figure 7A, control images). None of the WCSC exposures significantly altered these parameters (Figure 7A,B), indicating negligible morphological effects. In contrast, the positive control BAPTA-AM induced mitochondrial fragmentation and fission, characterized by rounded structures and disrupted networks, consistent with previous findings [35,36].

In line with these observations, WCSCs exerted minimal effects on the expression of Drp1, a central mediator of mitochondrial fission, or on its phosphorylation at Ser616 (Figure 7C) [37]. Similarly, the expression of Mfn1 and Mfn2, key regulators of mitochondrial fusion [38], remained unchanged following WCSC treatment.

### 3.5. Effect of WCSCs on mtROS Formation

**Figure 8:**
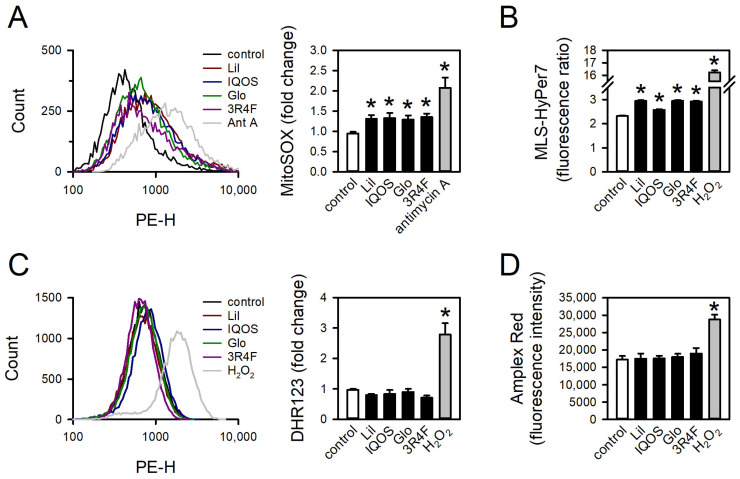
Increase in mtROS but not cytosolic ROS by WCSCs. Cells were treated with HTP- or 3R4F-WCSCs for 24 h. mtROS were detected with (**A**) MitoSOX Red or (**B**) MLS-HyPer7, and cytosolic ROS were measured with (**C**) DHR123 or (**D**) Amplex Red. (**A**,**C**) Flow cytometry histograms and quantification of mean fluorescence intensity normalized to untreated groups. Ant A: antimycin A. (**B**,**D**) Quantification of (**B**) MLS-HyPer7 ratio and (**D**) Amplex Red fluorescence intensity. Values are mean ± standard error [n = 7 for (**A**); n = 755, 876, 815, 712, 1088, and 166 for (**B**); n = 4 for (**C**); and n = 5 for (**D**)]. * *p* < 0.05 vs. control.

The effects of WCSCs on mtROS generation were evaluated using the mitochondrial superoxide indicator MitoSOX Red. All WCSC exposures significantly increased mtROS levels (Figure 8A). Comparable results were obtained with MLS-HyPer7, a genetically encoded biosensor specific for mtROS [39] (Figure 8B). In contrast, WCSCs did not elevate cytosolic ROS, as determined by DHR123 (Figure 8C) or Amplex Red (Figure 8D). The positive controls, antimycin A and hydrogen peroxide, significantly increased mtROS and cytosolic ROS, respectively, confirming assay validity [40]. These findings indicate that WCSC exposure selectively stimulates ROS generation within mitochondria without concomitant elevation of cytosolic ROS.

### 3.6. Effect of WCSCs on ATP Levels, Mitochondrial Respiration, and Membrane Potential

**Figure 9:**
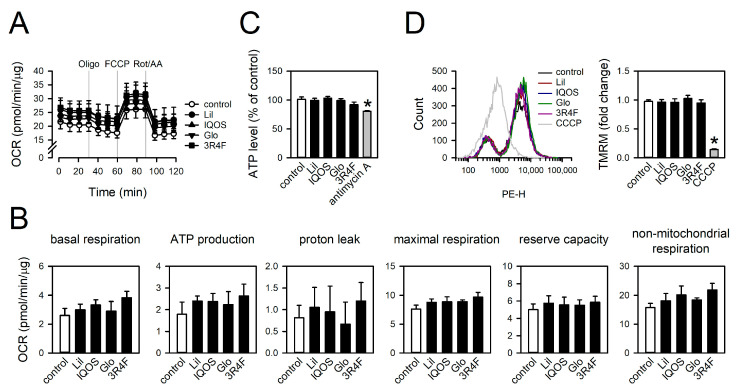
Effects of WCSCs on bioenergetic function, intracellular ATP levels, and mitochondrial membrane potential. Cells were treated with WCSCs for 24 h. (**A**) OCR traces representing bioenergetic functions. Oligo: oligomycin. (**B**) Quantification of mitochondrial respiration parameters, including basal respiration, ATP production, proton leak, maximal respiration, reserve capacity, and non-mitochondrial respiration. (**C**) Intracellular ATP levels normalized to untreated groups. (**D**) Flow cytometry histogram and normalized fluorescence intensity of TMRM-stained cells to assess mitochondrial membrane potential. Values are mean ± standard error [n = 8 for (**A**,**B**); n = 4 for (**C**), except CCCP-treated group (n = 3); and n = 9 for (**D**)]. * *p* < 0.05 vs. control.

Mitochondrial respiratory function was examined by measuring the OCR [41]. No significant differences in OCR traces were observed across WCSC-treated groups (Figure 9A). WCSCs did not induce significant changes in any respiratory parameters, including basal respiration, ATP-linked respiration, proton leak, maximal respiration, reserve capacity, or non-mitochondrial respiration (Figure 9B). Consistent with the minimal effects on bioenergetic function, intracellular ATP levels also remained unchanged, whereas the positive control antimycin A markedly reduced ATP content (Figure 9C).

The effects of WCSCs on mitochondrial membrane potential were evaluated using the fluorescent indicator TMRM, given that impairment of OXPHOS often results in membrane depolarization [42]. None of the WCSCs significantly altered mitochondrial membrane potential (Figure 9D). As expected, the positive control CCCP, a mitochondrial uncoupler, markedly reduced membrane potential [43]. Collectively, these results indicate that WCSCs increase mtROS without substantially disrupting mitochondrial respiration, ATP production, or membrane integrity.

## 4. Discussion

This study investigated the responses of bronchial epithelial cells to emissions from three HTPs (IQOS, Glo, and Lil) as well as the 3R4F reference cigarette, with particular emphasis on transcriptomic alterations. All WCSCs tested induced significant changes in gene expression. Overall, however, these alterations were less pronounced for HTP-WCSCs than for 3R4F-WCSC, and transcriptomic profiles were more similar among HTP-exposed groups than between HTP- and 3R4F-exposed groups (Figure 2 and Figure 3, Table 2). Although HTP-WCSCs affected diverse gene sets associated with distinct cellular functions, several pathways were commonly enriched across the different HTPs (Figure 4 and Figure 5). Among these, the mitochondrial OXPHOS pathway was one of the most consistently and significantly affected (Figure 5, Table 3). Both HTP-WCSCs and 3R4F-WCSC downregulated genes encoding OXPHOS complexes (Figure 6), leading to elevated mtROS with minimal effects on mitochondrial morphology or biogenesis (Figure 7, Figure 8 and Figure 9). These findings suggest that HTP use, irrespective of product type, can alter transcriptomic profiles in the respiratory epithelium and that mitochondria may represent a common toxicological target.

The present study examined three major HTPs. IQOS and Glo are the most widely marketed and studied globally, whereas Lil, produced by KT&G, is the leading HTP in Korea and is expanding internationally [44,45,46,47,48]. Although nicotine and TPM yields varied slightly among products (Table 1), their cellular effects converged on mitochondrial pathways, specifically OXPHOS gene downregulation and mtROS elevation. This convergence suggests that compositional or quantitative differences in emissions may not directly translate into differential cellular stress, underscoring the importance of overall chemical composition rather than individual constituents.

Determining appropriate exposure concentrations remains a challenge, particularly for complex mixtures such as HTP emissions or cigarette smoke. Nicotine is frequently used as a reference compound to standardize exposure across products, as it is the principal driver of dependence and a consistent marker of smoking intensity [49,50]. In this study, nicotine was used to normalize WCSC exposures across products and to facilitate direct comparison with 3R4F reference cigarettes. Because nicotine is a critical determinant of addiction and dependency, equivalent nicotine consumption was assumed to reflect comparable smoking intensity across product types. According to Health Canada Intense machine-smoking data, the nicotine concentration per puff of a 3R4F reference cigarette is approximately 0.199 mg, corresponding to ~3.62 µg/mL [31,32,33,51]. On this basis, a concentration of 3 µg nicotine/mL was employed in the present study. Preliminary experiments using lower concentrations (100 ng nicotine/mL WCSCs, reflecting a 1:30 dilution in the lung airways [15,52]) elicited only minimal transcriptomic changes. Thus, 3 µg nicotine/mL likely represents a threshold concentration sufficient to elicit measurable transcriptomic alterations in vitro. This nicotine-based approach offers advantages over TPM-based normalization, as TPM levels vary substantially among products. Notably, the concentration used here is considerably lower than that employed in many prior transcriptomic studies, which often relied on cytotoxic levels without appropriate justification [18,53,54]. While the clinical relevance of this chosen concentration remains uncertain, it is more directly linked to per-puff nicotine levels and therefore provides a more realistic representation of smoker exposure than most previous investigations.

The comparative enrichment analysis further highlights the distinct mitochondrial signature associated with HTP exposure (Figure 5). The OXPHOS pathway was consistently ranked among the top affected pathways in HTP-WCSC–exposed cells but showed a comparatively lower ranking in 3R4F-WCSC–treated cells (Figure 4, Table 3). Suppression of the OXPHOS pathway was stronger and more statistically significant in the HTP-treated groups (NES = −2.18 for Lil, −1.98 for IQOS, and −2.32 for Glo) than in 3R4F-treated cells (NES = −1.67) (Table 3). Indeed, the relative NES difference compared with 3R4F was greater for OXPHOS than for any other pathway (Figure 5). Despite these differences, both HTP- and 3R4F-WCSCs consistently downregulated OXPHOS-related genes and elevated mtROS levels. This lack of clear distinction suggests that even subtle effects of WCSC exposure may be sufficient to disrupt mitochondrial function. Because 3R4F-WCSC caused a broader and more extensive transcriptomic response—altering more than 1000 genes by ≥1.5-fold compared with fewer than 250 genes in each HTP-WCSC group (Table 2)—its impact on OXPHOS may be modulated by other transcriptomic changes. These findings are consistent with earlier transcriptomic studies reporting that HTPs generally induce fewer changes than conventional cigarettes [15,18,53], although direct comparisons remain limited by variability in experimental exposure conditions. Nonetheless, certain cellular processes may be more selectively or extensively affected by HTP exposure.

Cigarette emissions are capable of generating ROS both directly, through chemical interactions with aqueous media [55], and indirectly, by stimulating endogenous cellular sources such as NADPH oxidases [56,57]. Notably, in this study, WCSCs were completely removed after 24 h of exposure, prior to analysis. Thus, the observed elevation of mtROS reflects WCSC-induced genetic alterations rather than the direct chemical activity of WCSCs. Both HTP- and 3R4F-WCSCs suppressed expression of OXPHOS-related genes across multiple complexes, indicating widespread mitochondrial impairment. Interestingly, only mtROS levels were elevated, while mitochondrial morphology, OCR, ATP production, and membrane potential remained largely unaffected. These findings suggest that the exposure conditions employed were insufficient to induce collapse of mitochondrial integrity or overt mitochondrial dysfunction. Instead, mtROS elevation may represent an early biomarker of mitochondrial perturbation. Further studies are needed to determine whether prolonged or repeated exposures exacerbate these effects and ultimately lead to more pronounced mitochondrial dysfunction.

This study has several limitations. As with most in vitro experiments, reliance on an immortalized cell line limits direct extrapolation to in vivo airway physiology. Nevertheless, such models provide a controlled and practical system for elucidating novel mechanisms, including the mitochondrial effects of HTPs demonstrated herein. Moreover, exposures were conducted over a short-term, 24-h period, whereas chronic or repeated exposures more closely reflect habitual smoking. It remains uncertain, however, whether extended in vitro treatment across multiple passages can adequately model chronic human exposure, given the complex physiological interactions among diverse airway cell types. Finally, although we identified mtROS elevation as a consequence of OXPHOS suppression, downstream toxicological pathways—including redox signaling, antioxidant defenses, and inflammatory responses—were not examined. Future studies should address these aspects to clarify the clinical significance of HTP-induced transcriptomic and mitochondrial alterations.

## Figures and Tables

**Table 1:** TPM and nicotine concentrations in WCSCs.

	Lil	IQOS	Glo	3R4F
TPM	24.4	27.5	29.0	20.6
Nicotine	1.81	2.45	2.26	1.10

Values are shown as mg/mL.

**Table 2:** Quantitative analysis of DEGs in BEAS-2B cells exposed to HTP- and 3R4F-WCSCs.

	FC > 1.5		FC > 2	
	Up	Down	Up	Down
Lil	90	37	24	0
IQOS	104	67	32	1
Glo	113	112	36	4
3R4F	307	712	91	158

Values indicate the number of DEGs with *p* < 0.05 compared with the control.

**Table 3:** Significantly enriched Hallmark pathways in WCSC-exposed groups.

Pathway	Rank (NES)			
	Lil	IQOS	Glo	3R4F
Epithelial–mesenchymal transition	1 (−2.27)	1 (−2.43)	1 (−2.34)	1 (−2.43)
Oxidative phosphorylation	2 (−2.18)	6 (−1.98)	2 (−2.32)	17 (−1.67)
Unfolded protein response	3 (2.01)	9 (1.86)	12 (1.84)	12 (1.76)
Interferon-α response	4 (−1.94)	12 (−1.78)	20 (−1.70)	4 (−2.11)
Interferon-γ response	5 (−1.90)	14 (−1.69)	16 (−1.77)	8 (−1.89)
MTORC1 signaling	6 (1.85)	15 (1.68)	30 (1.61)	16 (1.68)
TGF-β signaling	7 (−1.84)	2 (−2.14)	4 (−2.06)	7 (−1.90)
Apical junction	8 (−1.83)	7 (−1.97)	5 (−1.99)	3 (−2.16)
Coagulation	9 (−1.80)	4 (−2.00)	3 (−2.06)	9 (−1.83)
MYC targets_v1	10 (−1.77)	17 (−1.66)	9 (−1.89)	15 (−1.69)
Apical surface	11 (−1.76)	8 (−1.88)	11 (−1.84)	5 (−2.01)
Notch signaling	12 (−1.74)	10 (−1.82)	6 (−1.97)	false
Fatty acid metabolism	13 (−1.67)	16 (−1.67)	7 (−1.97)	false
Complement	14 (−1.56)	13 (−1.74)	21 (−1.69)	19 (−1.65)
Myogenesis	15 (−1.55)	28 (−1.40)	19 (−1.73)	11 (−1.80)
Cholesterol homeostasis	16 (−1.54)	11 (−1.80)	17 (−1.74)	18 (−1.65)
Peroxisome	17 (−1.53)	23 (−1.52)	23 (−1.66)	false
Glycolysis	18 (−1.51)	19 (−1.59)	8 (−1.96)	25 (−1.57)
Androgen response	false	3 (−2.09)	13 (−1.82)	13 (−1.73)
UV response_down	false	5 (−1.99)	29 (−1.62)	2 (−2.27)
Angiogenesis	false	18 (−1.61)	−14 (1.81)	6 (−1.94)
TNFα signaling via NF-κB	false	20 (−1.57)	15 (−1.79)	31 (−1.42)
Inflammatory response	false	21 (−1.57)	27 (−1.63)	21 (−1.58)
WNTβ catenin signaling	false	22 (−1.57)	28 (−1.63)	false
G2M checkpoint	false	24 (−1.46)	32 (−1.50)	28 (−1.55)
Estrogen response_late	false	25 (−1.45)	22 (−1.67)	14 (−1.70)
Estrogen response_early	false	26 (−1.45)	31 (−1.53)	24 (−1.57)
KRAS signaling_up	false	27 (−1.45)	24 (−1.65)	10 (−1.83)
p53 pathway	false	29 (1.37)	false	false
Hypoxia	false	30 (−1.36)	10 (−1.88)	23 (−1.57)
Reactive oxygen species pathway	false	false	18 (−1.74)	false
Apoptosis	false	false	25 (−1.64)	30 (−1.47)
Adipogenesis	false	false	26 (−1.64)	false
Allograft rejection	false	false	33 (−1.40)	20 (−1.62)
DNA repair	false	false	34 (−1.34)	false
Xenobiotic metabolism	false	false	false	22 (1.57)
IL6-JAK-STAT3 signaling	false	false	false	26 (−1.56)
E2F targets	false	false	false	27 (−1.56)
KRAS signaling_down	false	false	false	29 (−1.53)
Mitotic spindle	false	false	false	32 (−1.40)

Significantly enriched pathways (adjusted *p* < 0.05) identified by GSEA are listed in descending order of |NES|, beginning with those significant in Lil, and subsequently including additional significant pathways from IQOS, Glo, and 3R4F groups. false: adjusted *p* > 0.05.

## Data Availability

The data supporting the findings of this study are available from the corresponding authors upon reasonable request.

## Abbreviations

The following abbreviations are used in this manuscript:

HTPs	Heated tobacco products
WCSCs	Whole cigarette smoke condensates
OXPHOS	Oxidative phosphorylation
ROS	Reactive oxygen species
KT&G	Korea tomorrow & global
DHR123	Dihydrorhodamine 123
TMRM	Tetramethylrhodamine methyl ester
Amplex Red	10-Acetyl-3,7-dihydroxyphenoxazine
BCA	Bicinchoninic acid
RPMI	Roswell Park Memorial Institute
FBS	Fetal bovine serum
HBSS	Hank’s balanced salt solution
Drp1	Dynamin-related protein 1
Mfn1	Mitofusin-1
Mfn2	Mitofusin-2
HRP	Horseradish peroxidase
CCCP	Carbonyl cyanide m-chlorophenyl hydrazone
RIPA	Radioimmuno-precipitation assay
CSE	Cigarette smoke extract
TPM	Total particulate matter
SDS	Sodium dodecyl sulfate
PCR	Polymerase chain reaction
GSEA	Gene set enrichment analysis
FDR	False discovery rate
NES	Normalized enrichment score
mtROS	Mitochondrial ROS
OCR	Oxygen consumption rate
FCCP	Carbonyl cyanide-4-(trifluoro-methoxy) phenylhydrazone
PCA	Principal component analysis
DEG	Differentially expressed gene
MsigDB	Molecular Signatures Database
